# Cost-effectiveness simulation and analysis of colorectal cancer screening in Hong Kong Chinese population: comparison amongst colonoscopy, guaiac and immunologic fecal occult blood testing

**DOI:** 10.1186/s12885-015-1730-y

**Published:** 2015-10-15

**Authors:** Carlos KH Wong, Cindy LK Lam, YF Wan, Daniel YT Fong

**Affiliations:** 1 Department of Family Medicine and Primary Care, The University of Hong Kong, 3/F, Ap Lei Chau Clinic, 161 Ap Lei Chau Main Street, Ap Lei Chau, Hong Kong, Hong Kong; 2 School of Nursing, The University of Hong Kong, Hong Kong, Hong Kong

**Keywords:** Cost-effectiveness, Colorectal cancer, Fecal occult blood testing, Colonoscopy, Mass screening

## Abstract

**Background:**

The aim of this study was to evaluate the cost-effectiveness of CRC screening strategies from the healthcare service provider perspective based on Chinese population.

**Methods:**

A Markov model was constructed to compare the cost-effectiveness of recommended screening strategies including annual/biennial guaiac fecal occult blood testing (G-FOBT), annual/biennial immunologic FOBT (I-FOBT), and colonoscopy every 10 years in Chinese aged 50 year over a 25-year period. External validity of model was tested against data retrieved from published randomized controlled trials of G-FOBT. Recourse use data collected from Chinese subjects among staging of colorectal neoplasm were combined with published unit cost data ($USD in 2009 price values) to estimate a stage-specific cost per patient. Quality-adjusted life-years (QALYs) were quantified based on the stage duration and SF-6D preference-based value of each stage. The cost-effectiveness outcome was the incremental cost-effectiveness ratio (ICER) represented by costs per life-years (LY) and costs per QALYs gained.

**Results:**

In base-case scenario, the non-dominated strategies were annual and biennial I-FOBT. Compared with no screening, the ICER presented $20,542/LYs and $3155/QALYs gained for annual I-FOBT, and $19,838/LYs gained and $2976/QALYs gained for biennial I-FOBT. The optimal screening strategy was annual I-FOBT that attained the highest ICER at the threshold of $50,000 per LYs or QALYs gained.

**Conclusion:**

The Markov model informed the health policymakers that I-FOBT every year may be the most effective and cost-effective CRC screening strategy among recommended screening strategies, depending on the willingness-to-pay of mass screening for Chinese population.

**Trial registration:**

ClinicalTrials.gov Identifier NCT02038283

**Electronic supplementary material:**

The online version of this article (doi:10.1186/s12885-015-1730-y) contains supplementary material, which is available to authorized users.

## Background

Accumulated evidence suggested that screening by fecal occult blood test (FOBT) is effective in reducing annual CRC incidence and annual mortality [1]. Colonoscopy and flexible sigmoidoscopy are alternative strategies recommended for CRC screening [2] whereas population-based case–control studies in the US have shown considerable reduction in annual mortality from colonoscopy screening [3, 4]. Ideally randomized controlled trial (RCT) provides direct empirical evidence of comparative effectiveness of CRC screening strategies. To capture such long-term CRC risk, previous RCTs were designed to randomly allocate subjects into regular FOBT screening group and no screening group lasted for at least 10 years [5–9]. A RCT of assessing the comparative effectiveness of one-time colonoscopy and I-FOBT is on-going and expected to be completed in 2021 [10]. To strike a balance between costs and effectiveness incurred by CRC screening, cost-effectiveness analysis (CEA) provides decision and justification for efficient resource allocation under a fixed budget constraint.

Cost-effectiveness modeling on the US population has shown that annual FOBT plus 5-yearly sigmoidoscopy under full compliance rate [11] and colonoscopy every 10 years [12] are the most cost-effective in terms of life years (LYs) gain for an average-risk population. A study on the Hong Kong population found that FOBT and colonoscopy had an incremental cost of US$6222 and US$7211 per life year gained compared to no screening, respectively [13]. Modeling by Woo et al. suggested that Chinese women from age 50 to 75 years by colonoscopy every 10 years compared to no screening had an incremental cost of US$55545 per disability-adjusted life years averted [14]. However, the National Centre for Clinical Excellence (NICE) recommended that quality of life measured by a valid preference-based measure of health should be incorporated into the outcome measure of effectiveness, to so called quality adjusted life years (QALYs), in analysis of an medical intervention [15]. The QALYs is the outcome measure of effectiveness on which to incorporate both morbidity and mortality of patients. UK studies estimated the incremental cost of biennial FOBT compared to no screening to be below £3000 per QALYs, and thus biennial FOBT alone was the most cost-effective screening strategy [16, 17]. The optimal screening strategies in the USA and Canada become colonoscopy every 10 years [18, 19]. However, projected results of multiple studies may not be extrapolated to the Chinese population.

Although the CRC incidence rate of the Chinese populations is approaching those of developed countries [20], there is no agreed policy on CRC screening for the Chinese population in Hong Kong or mainland China. No CEA of CRC screening in terms of QALYs gain has ever been done on Chinese populations. Most CEA on FOBT were based on G-FOBT, evaluation of the more accurate but more expensive I-FOBT is warranted. Therefore, the aim of paper was to evaluate the in-depth cost-effectiveness analysis of colorectal cancer screening strategies from the healthcare service provider perspective in Hong Kong, China. The specific objectives were 1) to determine the expected life years gained from the reduction in the incidence and mortality rates of CRC for each CRC screening strategy, 2) to determine the QALY gained from each CRC strategy by combining the preference value with life years gained, and 3) to identify the most cost-effective CRC screening strategy and to determine the incremental cost per additional QALY gained compared to no screening, by Markov modeling.

## Methods

Ethical approval was obtained from The University of Hong Kong/Hospital Authority Hong Kong West Cluster institutional review board (HKU/HA HKW IRB #UW 09–391), and this trial was registered with Hong Kong Clinical Trial Register (#HKCTR-973).

### Model overview

**Fig. 1:**
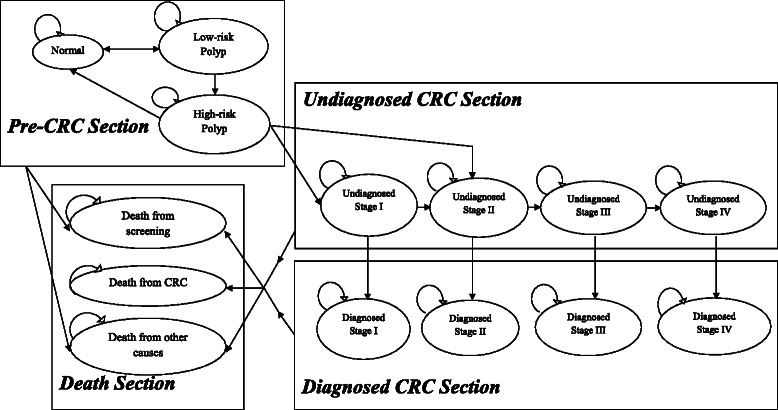
Annual Transition of health states in Markov Modelling

Six screening strategies for colorectal adenomas and CRC were compared in cost and effectiveness under a decision analytic model based on a state-transition Markov process [21]. A hypothetical static cohort of 100,000 persons from 50-year-old Hong Kong population entered the model and their health histories were simulated by sex until 75 years old. Under the model framework, each person had an initial health state based on the distribution of colorectal adenomas [22]. The natural history of colorectal neoplasms (CRN) was reflected on the model via the transitions between different health states and the mortalities (Fig. 1). Superimposed on the natural history were the screening interventions and subsequent colonoscopic surveillance after polyp removal or stage-specific treatment upon the detection of a CRC.

### Natural history

The key feature of the model was the health states of CRN which were divided into four sections:

- the Pre-CRC section included “Normal colonic epithelium”, “Low-risk polyps” and “High-risk polyps”;
- the Undiagnosed CRC section consisted of “Undiagnosed Stage I CRC”, “Undiagnosed Stage II CRC”, “Undiagnosed Stage III CRC” and “Undiagnosed Stage IV CRC”;
- the Diagnosed CRC section was comprised of “Diagnosed Stage I CRC”, “Diagnosed Stage II CRC”, “Diagnosed Stage III CRC” and “Diagnosed Stage IV CRC”;
- the Death section was divided into “Death from CRC”, “Death from screening complications” and “Death from other causes”.

According to the screening surveillance guideline [23], low-risk polyps are defined as ≤2 adenomas or 3–4 adenomas which are < 1 cm while high-risk polyps are defined as ≥5 adenomas or ≥3 adenomas of which at least one is ≥ 1 cm. The health states of CRC were classified by the American Joint Committee on Cancer staging system [24].

All health states were modelled as Markov states with 1-year cycle. A person would transit to a different health state or remain at its current health state at the end of every 1-year period in the Markov process [21]. With different transition probabilities employed to link a health state to the others, the model tried to capture the essence of the natural history of CRN. All health states were at risk to the progression to a more advanced disease stage or death, but they were prohibited from returning to the former health states except that low-risk and high-risk polyps patients could recover and return to normal colonic epithelium after polyp removal with polypectomy. It was assumed that normal colonic epithelium and low-risk polyps were at no risk of progression to CRC in a 1-year cycle, the transition probability between normal colonic epithelium and low-risk polyps was taken from a previous study [16] which summarized the incidence rates of adenomas within the average risk population. The annual probabilities that low-risk polyps develop into high-risk polyps, or high-risks polyps develop into non-metastatic CRC were taken from a cost-effectiveness analysis [25]. CRC patients could either be clinically undiagnosed or diagnosed. Undiagnosed CRC were at risk of progression to more advanced stages of CRC and mortality from CRC or other causes. Each year those CRC undiagnosed patients had a certain probability of symptomatic presentation [26], in which case they were assumed to consult a physician and to have the diagnosis confirmed by colonoscopy. Another condition of CRC diagnosis was detection of the malignancy by screening interventions. When they were diagnosed to have CRC, they would receive specific assessments and treatments according to disease stages. It was assumed that the risk of disease progression was eliminated once the CRC was diagnosed and treated and they would remain in the same health state but they were still at risk of mortality from CRC or other causes.

The most severe health states were the three causes of death, described as the absorbing stages in the terminology of Markov processes [21]. Death from CRC meant dying from clinical complications of CRC. The annual CRC mortalities by stage of disease were extracted from a Chinese study which was based on the Hong Kong Cancer Registry in 2007 [13]. There had been no mass CRC screening programmes in Hong Kong so it was valid to use the general population CRC mortality data to represent the natural history that was not modified by screening interventions. Death from screening complications was specifically formulated to reflect the risk of mortality from serious complications of bleeding or perforation in endoscopic procedures. However, mortalities from other complications (e.g. drug anaphylaxis) were not considered in this model. Death from other causes mirrored the mortalities from all possible causes apart from those related to CRC and screening complications. The corresponding annual mortality from causes other than CRC and screening complications was estimated by the non-CRC mortality, which was derived by subtracting the CRC mortality by sex and quinqueenial age groups (e.g. 50–54, 55–59, etc.) [27] from the all-cause mortality by sex and age which was quoted from the Hong Kong Life Table in 2007 [28]. The natural history parameters for the model are shown in Additional file 1.

### Screening strategies

The screening strategies were designed based on 3 core screening interventions, i.e. G-FOBT, I-FOBT and colonoscopy, with different screening periods. Six commonly used strategies were identified from a review of the US national guidelines [2, 29], previous cost-effectiveness analysis studies [16, 30], population-based screening programmes [6–8, 31–35] and local studies [36]. Among them, 5 were single-intervention strategies, and a no screening strategy functioned as a control. The repeated period of screening is 1 or 2 year (s) for G-FOBT/I-FOBT, and 10 years for colonoscopy. The strategies were listed below:

i. no screening
ii. annual G-FOBT (Hemoccult-II SENSA, Beckman Coulter, Inc., California, USA)
iii. annual I-FOBT (actim Fecal Blood, Medix Biochemica, Finland)
iv. biennial G-FOBT (Hemoccult-II SENSA, Beckman Coulter, Inc., California, USA)
v. biennial I-FOBT (actim Fecal Blood, Medix Biochemica, Finland)
ix. ix. colonoscopy every 10 years

With the one-sample per screening round, people who had positive G-FOBT or qualitative I-FOBT result were assumed to proceed immediately to a colonoscopy to confirm the result. Polypectomy would be undertaken once any polyp was found on colonoscopy. After polyp removal, a surveillance colonoscopy was assigned to the patient every 5 years if the polyp was of low-risk and every 1 year if of high-risk. If CRC rather than polyp was detected, the patient transacted to a CRC state and received specific assessment and treatment according to the disease stage of CRC.

### Diagnostic performance of the screening tests

The performance of the screening tests was determined by the sensitivities and specificities in detecting adenomatous polyps and cancers. Sensitivities and specificities associated with G-FOBT and I-FOBT were based on the results of two local Hong Kong studies [22, 37]. The sensitivity and specificity associated with colonoscopy were assumed to be 100 % although there were no research data on the true accuracy of colonoscopy [38]. When accessing the diagnostic performance of a screening test, it is important to take into account the possible serious complications. This consideration is irrelevant to G-FOBT and I-FOBT as these are no complications associated with those tests. For colonoscopy, the major severe complications are bleeding and perforation. The probabilities of bleeding and perforation for colonoscopy as well as the mortalities from these complications were estimated from the data of several overseas studies since local data were not available [39–46]. Additional file 1 shows the performance characteristics of the G-FOBT, I-FOBT and colonoscopy.

### Screening participation

Screening interventions assigned to a person are not mandatory. One has the right to refused attending a screening even if it was scheduled with free of charge. This important fact affects the ‘efficacy’ of a screening intervention significantly. Our model first assumed that a person had a constant probability to participate in any kind of screening intervention each time it was assigned to the person, independent of the individual’s past history of participation in screening or surveillance for CRC. A constant compliance rate of 60 % was assumed for all screening interventions involved in the 6 screening strategies in the base-case scenario [11]. For the follow-up colonoscopy after positive test result in the initial screening or the surveillance colonoscopy after polyp removal or CRC diagnosis, a high compliance rate of 80 % was assumed [11]. For those patients who had symptomatic presentation of CRC, it was assumed a full compliance on the colonoscopy screening arrangement after physician consultation, and the patient withdrew from the screening strategy originally arranged. Additional file 1 shows the compliance rate on G-FOBT, I-FOBT and colonoscopy.

### Model validation

External validity of our model was accessed by comparing the model outcomes with the study results from published clinical studies which were anticipated to be consistent with the model findings [47]. The cohort size and patient characteristics were modified to replicate that of the population or sample of the data source applied. Our model was initiated by obtaining similar outcome measures as the published data so that head-to-head comparisons were made. One criterion of CRC mortality rate reduction was assessed under this framework. Taking the ratio of CRC mortality rates, the reduction in mortality rate of a screening strategy from the other competing strategies was calculated. The reduction in CRC mortality for G-FOBT reported by randomized controlled trials [5, 7, 8] was compared with our model results (Additional file 1). The reductions for colonoscopy reported by case–control studies [3, 4, 48] were incomparable with our model results for colonoscopy every 10 years because the previous studies reported irregular screening interval for repeated colonoscopy.

Additional file 1 shows a comparison of model-anticipated CRC mortality rates reduction compared with equivalent previous studies estimate. Our Markov model appeared to provide excellent fit of CRC mortality reduction by biennial G-FOBT against results from Funen trial data [7] whilst the model reported a reasonable fit against reduction in CRC mortality reported with Nottingham and Minnesota screening trials [5, 8]. Comparison indicated an acceptable model validity of predicting the reduction in CRC mortality from annual and biennial G-FOBT.

### Model outcomes

#### Costs outcomes

The perspective of health service provider was adopted when evaluating the costs for the CRN care, so only direct medical costs were incorporated to the model. The costs were divided into three groups according to the period of the diagnosis of CRN: Pre-diagnosis, First year of diagnosis, and Subsequent years of diagnosis. Costs of cancer care were primarily allocated to the initial phase (first year of diagnosis) as well as the continuing and terminal phases (subsequent years of diagnosis) of care after the diagnosis of CRC. Costs for the terminal phase of care were assumed to be priced in the same way as the continuing phase. In the pre-diagnosis phase, only screening of CRN contributed to the costs but the treatment was included. The stage-specific costs for CRN care were derived from the usage data of the relevant screening tests though the modeling. Costs for the initial phase of care were extracted from a Hong Kong study [49] which summarized the local direct medical costs for each health state of CRN, while that for the subsequent years of diagnosis were drawn from the guideline of polyps surveillance after polyps removal [23] and the cancer treatment protocol on the recommended use of medical services following surgical operation [50]. Unit costs estimates associated with the screening tests and the outpatient follow-up in specialist clinics (including basic investigation tests such as Chest X-rays and laboratory tests) were based on the published data from the Government Gazette [51]. The costs of the screening complications were derived from a previous cost-effectiveness analysis modeled on Chinese population [13]. Local costs evaluated in Hong Kong dollar (year 2009 values) were converted to US dollar at the pegged exchange rate of USD 1 = HKD 7.8. Unit costs of the service components and the stage-specific costs of initial care are shown in Additional file 1. Direct medical costs of care related to CRN were accumulated for each cycle over the screening period of 25 years. The technique of half-cycle correlation was applied to give more accurate measures of the costs [21]. The lifetime medical costs per person for all screening strategies were the outcome of cost measure. All the costs were discounted by an annual rate of 3.5 % as recommended by the guidance of NICE [15].

#### Effectiveness outcomes

Two effectiveness outcomes were assessed by the Markov model: the LYs and QALYs. The life expectancy of each cohort under a particular screening strategy was calculated. The QALYs are generated by adjusting the LYs according to a preference-based measure of health-related quality of life. The LYs and QALYs gained of a screening strategy from the other was computed by taking the difference of the life expectancies and quality-adjusted life expectancies of the two strategies, respectively.

Utility scores of each health state of the CRN patients defined in our model was associated with a constant utility score, representing “death” of 0 and “perfect health” of 1. Provided that the scoring algorithm for utility score is culture-specific, we adopted the utility input from an existing scoring algorithm developed based on local population. To date, Chinese version of SF-6D with the Hong Kong Chinese population based scoring algorithm [52, 53] made available to compute the SF-6D utility scores whilst scoring algorithms for other utility metrics such as EQ-5D did not. Moreover, the SF-6D score was shown to be responsive to change in Hong Kong Chinese population [54]. Hence, the estimates of the stage-specific utility scores were adopted from a study [55]. People who had normal colorectal epithelium were assumed to have perfect health with utility score of 1 [18]. Half-cycle correlation was used again for the measures of LYs and QALYs [21], and they were discounted as the same rate as the costs, i.e. 3.5 % annually [15].

#### Cost-effectiveness analysis

Core outcome of the cost-effectiveness analysis was the incremental cost-effectiveness ratio (ICER) which was calculated by dividing the incremental cost (∆C) by the incremental effectiveness (∆E) in terms of LYs or QALYs gained for a particular screening strategy compared to other less effective screening strategy. The cost-effectiveness analysis was executed by the comparison of the ICER values of different screening strategies.

The dominated and extended dominated strategies were reported on the figures [56]. By definition, the strategy is dominated if it is less effective and most expensive than one of the competing strategies. The strategy is regarded as extended dominance if it is less effective and had a higher ICER than one of the competing strategies. The line connecting the strategies which were not dominance and extended dominance formed the efficiency frontier [57]. The ICER values of any two adjacent strategies on the efficiency frontier were determined. For a given ceiling ratio of λ, which is the maximum amount of willingness-to-pay per effectiveness gain [58], the optimal strategy was defined as the one with the highest ICER value below λ, compared to the next less effective strategy on the efficiency frontier. Accumulative In current study, the ceiling ratio was defined at a threshold of US$50,000 per effectiveness gained [19, 59–63].

#### Sensitivity analysis

Deterministic (univariate and multivariate) and probabilistic sensitivity analysis (PrSA) were performed to explore the stochasticity and uncertainty on the model parameters and outputs. Univariate sensitivity analysis for the ICER of any two non-dominated strategies on the efficiency frontiers was conducted on the major screening based variables which included compliance rates of screening, follow-up and surveillance colonoscopy and performance characteristic of each screening strategy. In addition, the utilities of the different health states, the disease stage-specific treatment costs, the transition probabilities, the CRC mortalities, the probabilities of symptomatic presentation, and annual discount rate were also included in the analysis. The cut-off values used in the univariate sensitivity analysis were the minimum and maximum values extracted from the literature. In case no such information was available, 95 % confidence limits or values suggested by clinical experts were used. Multivariate sensitivity analysis varied difference sets of utility scores for health states reported in previous models [16, 19, 64].

PrSA was conducted finally to achieve a full examination of the uncertainty involved in the model parameters and consequently the model outputs. All the parameters except the time horizon and the discount rate were associated with a probability distribution. A Monte Carlo simulation was carried out to randomly draw from those distributions for 10,000 iterations. CRC Mortality rate from cancer registry were excluded from the PrSA as parameter uncertainty is small. The probability distributions with associated distribution parameters are displayed in Additional file 1. Cost parameters were assigned to be log-normally distributed whilst probability, rate and utility parameters were assigned to beta-distribution [65]. The cost and effectiveness (in QALYs or LYs) for a strategy compared to no screening were computed for the 10,000 iterations. The cost-effectiveness acceptability curve [57] was then constructed to demonstrate the probability of being cost-effective for each strategy in the 10,000 iterations at each level of the ceiling ratio.

The main computational tool we used to perform the cost-effectiveness analysis was TreeAge Pro Suite 2009 Release 1.0.2 (TreeAge Software, Inc., Williamstown, MA, US). The Microsoft Excel 2010 was used for supplementary analysis and graphical production.

## Results

### Base-case scenario

Table 1 shows the incremental cost of a screening strategy from the other competing strategies, ranked in the ascending order of effectiveness. With additional work-up due to screening, every screening intervention was more expensive than no screening. The most expensive strategy was annual G-FOBT costing $2853 more compared to no screening. Apart from no screening, the cheapest strategy was biennial G-FOBT which costs $1681 more than no screening. Annual FOBT screening did cost more than colonoscopy and biennial FOBT screening. By convention, every CRC screening strategy extended life expectancy and quality adjusted life expectancy. Annual I-FOBT was the most effective CRC screening strategy, in which provided 0.12305 LYs and 0.80121 QALYs compared to no screening. Colonoscopy every 10 years gained more life expectancies than the biennial G-FOBT while colonoscopy every 10 years averted more quality adjusted life expectancies than biennial G-FOBT over a simulated period of 25 years.

**Table 1:** Cost, LYs and QALYs per person for each screening strategy, and the incremental cost, LYs and QALYs of a screening strategy compared with no screening

Strategy^a^	No Screening	Biennial G-FOBT	Annual G-FOBT	Colonoscopy every 10 years	Biennial I-FOBT	Annual I-FOBT
Cost ($USD) per person	2541	4221	5394	4752	4542	5068
Incremental cost (∆C, $) compared with no screening	-	1681	2853	2212	2001	2528
Expected LYs per person	15.6420	15.6862	15.7104	15.7385	15.7429	15.7650
Incremental LYs compared with no screening	-	0.0443	0.0684	0.0965	0.1009	0.1231
Expected QALYs per person	14.7479	15.0687	15.2339	15.3586	15.4203	15.5491
Incremental QALYs compared with no screening	-	0.3207	0.4860	0.6106	0.6724	0.8012

Note: G-FOBT, Guaiac fecal occult blood testing; I-FOBT, immunologic fecal occult blood testing

^a^Sort by ascending order of effectiveness

**Fig. 2:**
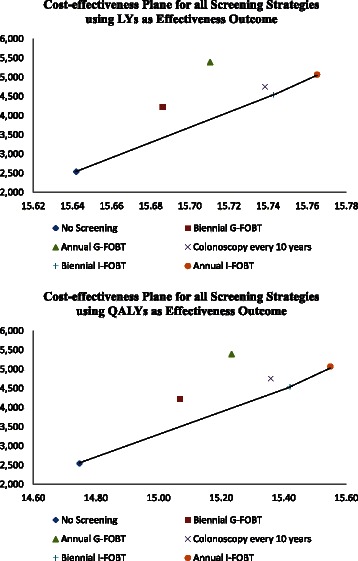
Cost-effectiveness Plane for all the Six Screening Strategies using LYs (Upper) and QALYs (Lower) as Effectiveness Outcome

Table 2 shows the incremental cost-effectiveness ratio in term of cost per LYs and cost per QALYs of a screening strategy from the other competing strategies, respectively. The plots of the cost-effectiveness plane against the two effectiveness measures of LYs and QALYs respectively for all the six screening strategies are presented in Fig. 2. Taking account of life expectancy only and quality of life adjustment, biennial G-FOBT was extended dominated because it was slightly less effective than biennial I-FOBT, and had higher ICER ($37,985/LYs vs $19,838/LYs; $5240/QALYs vs $2976/QALYs) than biennial I-FOBT relative to no screening. Strategies of colonoscopy every 10 years and annual G-FOBT were dominated with lower effectiveness and higher costs. All I-FOBT screening remained more effective and cost-effective than colonoscopy and G-FOBT screening.

**Table 2:** The ICER in terms of $/LYs or $/QALYs of a Screening Strategy from the Other Competing Strategies

Strategy^a^	ICER	Biennial G-FOBT	Annual G-FOBT	Colonoscopy every 10 years	Biennial I-FOBT	Annual I-FOBT
No Screening	$/LYs	37,985	41,688	22,913	19,838	20,542
	$/QALYs	5240	5871	3622	2976	3155
Biennial G-FOBT	$/LYs		48,461	10,154	5657	10,747
	$/QALYs		7096	1831	911	1763
Annual G-FOBT	$/LYs			Dominance^b^	Dominance^b^	Dominance^b^
	$/QALYs					
Colonoscopy every 10 years	$/LYs				Dominance^b^	11,916
	$/QALYs					1659
Biennial I-FOBT	$/LYs					23,742
	$/QALYs					4087

Note: G-FOBT, Guaiac fecal occult blood testing; I-FOBT, immunologic fecal occult blood testing; ICER, Incremental cost-effectiveness ratio

^a^Sort by ascending order of effectiveness

^b^Annual G-FOBT was dominated by colonoscopy every 10 years and I-FOBT every 1 or 2 year(s) whereas colonoscopy every 10 years was dominated by biennial I-FOBT

The ICERs for annual I-FOBT, colonoscopy every 10 years and annual G-FOBT presented $24,608/QALYs, $3155/QALYs, $3622/QALYs and $5871/QALYs gained when competing with no screening respectively. Hence, the ICERs were far below from the willingness-to-pay threshold of approximately $50,000/QALYs gained.

Table 3 gives the ranges of λ which the optimal strategy varies from one range to another competing strategy. Biennial I-FOBT was the optimal screening strategy for a range of $19,838-$23,742/LYs ($2976-$4087/QALYs) whilst annual I-FOBT was the optimal strategy in threshold of more than $23,742/LYs or $4087/QALYs. Default no screening would be the most optimal screening strategy for CRC in the range of ceiling ratio between zero and $19,838/LYs (or $2976/QALYs). As a consequence, annual I-FOBT was the optimal strategy with an ICER closet to $50,000 per LYs as well as per QALYs.

**Table 3:** Optimal strategy according to the Ceiling Ratio in Base-case and Multivariate Scenarios

	Optimal Strategy					
Ceiling Ratio	No Screening	Biennial G-FOBT	Annual G-FOBT	Colonoscopy every 10 years	Biennial I-FOBT	Annual I-FOBT
*In term of LYs*						
Base-case scenario						
[0, 19,838]		Extended Dominance	Dominance	Dominance	(19,838, 23,742]	(23,742, +∞)
Non-discounted Scenario (Discount Rate = 0 %)						
[0, 14,681]		Extended Dominance	Dominance	Dominance	(14,681, 15,856]	(15,856, +∞)
*In term of QALYs*						
Base-case scenario						
[0, 2976]		Extended Dominance	Dominance	Dominance	(2976, 4087]	(4087, +∞)
Non-discounted Scenario (Discount Rate = 0 %)						
[0, 2510]		Extended Dominance	Dominance	Dominance	(2510, 3294]	(3294, +∞)
Ramsey’s Utility Set Scenario (Cancer free = 1.00; S1/S2 = 0.90; S3 = 0.80; S4 = 0.76)						
[0, 12,294]		Extended Dominance	Dominance	Dominance	(12,294, 15,279]	(15,279, +∞)
Ness’s Utility Set Scenario (Cancer free = 0.91; S1 = 0.74; S2 = 0.70; S3 = 0.50; S4 = 0.25)						
[0, 9278]		Extended Dominance	Dominance	Dominance	(9278, 11,803]	(11,803, +∞)
Sharp’s Utility Set Scenario (Cancer free = 0.94; S1/S2/S3/S4 = 0.80)						
[0, 12,505]		Extended Dominance	Dominance	Dominance	(12,505, 15,460]	(15,460, +∞)

Note: G-FOBT, Guaiac fecal occult blood testing; I-FOBT, immunologic fecal occult blood testing; ∞, infinity

### Sensitivity analysis

Results of the one-way sensitivity analysis for ICER for the comparisons amongst annual I-FOBT, biennial I-FOBT and no screening were described below. The most sensitive collection of clinical parameters was the natural history parameters representing the annual transition probabilities between health states. Varying unit costs in resource used in care of CRN and utility scores for health state had limited impact on the cost-effectiveness comparing amongst non-dominated strategies. However, the specificity of I-FOBT was the most influential parameter when annual I-FOBT was compared with biennial FOBT. Decreased specificity of I-FOBT was associated with an increased in ICER for annual I-FOBT compared with biennial I-FOBT.

**Fig. 3:**
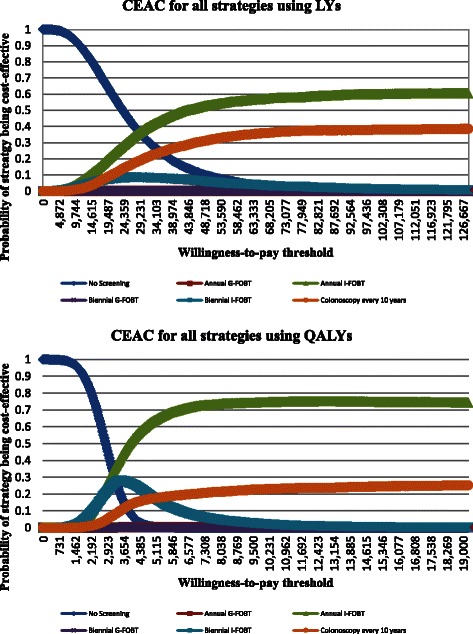
Cost-effectiveness Acceptability Curve (CEAC) in term of LYs (Upper) and QALYs (Lower) for all Strategies in Probabilistic Sensitivity Analysis

Figure 3 shows the results of PrSA using the cost-effectiveness acceptability curve. Results demonstrated that no strategy had a probability to be optimal higher than 60 % at a ceiling ratio of $50,000 per LY gained. Given a maximum acceptable ceiling ratio of $7000 per QALY gained, the probability that annual I-FOBT is cost-effective compared with other screening strategies exceeded 70 % but the probability that colonoscopy every 10 years is cost-effective was about 20 %. The probability of annual I-FOBT and colonoscopy every 10 years being cost-effective converged to 75 and 25 %, respective, if the maximum acceptable ceiling ratio increased to $50,000 per QALY gained.

## Discussion

The present paper demonstrated the cost-effectiveness of CRC screening using Chinese data on evaluating the most cost-effective strategy based on two cost-effectiveness outcomes, cost per LYs and QALYs gained. The model compared six strategies for CRC screening currently implemented by population-based screening programmes, and recommended by international guidelines and previous studies. The fact that no uniform screening strategies is currently implemented in healthcare provider setting in Hong Kong and mainland China unleashes the comparative cost-effectiveness of no screening relative to other screening strategies in Chinese populations. In this model, no screening interventions exceeded the threshold of US$50,000/QALYs gained. Given the low ICER for every screening intervention, additional benefits provided by CRC screening appears to be cost-effective compared to no screening in case when the greater willingness-to-pay for screening was possessed by health policymakers. The cost-effective plane provided the judgment that annual G-FOBT and colonoscopy every 10 years were dominated by I-FOBT screening, irrespective of annual or biennial repeated period. Either one of I-FOBT screenings was more cost-effective than all competing screening strategies for a given ceiling ratio of more than US$19,838/LYs or US$2976/QALYs gained. In other words, no screening was favorable compared to CRC screening at a ceiling ratio of not more than US$19,838/LYs or US$2976/QALYs gained. The annual I-FOBT screening was found to be preferable for a ceiling ratio of US$23,742/LYs or US$4087/QALYs gained.

The model provided evidence that the I-FOBT screening strategy, with superiority in sensitivity and specificity, was more cost-effective than G-FOBT, which was in line with the majority of modeling studies comparing between guaiac and immunologic testing, as indicated by the US [12, 63] and other countries [18, 66, 67]. Considering the differences between screening strategies under the same screening interval, the annual I-FOBT dominated the annual G-FOBT. Alternatively, the biennial G-FOBT was extended dominance by colonoscopy every 10 years, which was discordant with the recent Australian and French studies [68, 69] comparing between these two strategies, concluding that biennial G-FOBT was more cost-effective than colonoscopy every 10 years. However, in earlier Australian reports [70, 71], the ICER for colonoscopy was lower than that for biennial G-FOBT, indicating the extended dominance of G-FOBT.

To our knowledge, six models were built with the utility input for health states. Three models [16, 18, 60, 72] obtained the health preference scores for each CRC health states from a study [73] on the basis of direct elicitation using a standard gamble exercise, while the others [19, 64] used the health preference scores for each CRC stage estimated from Health Utility Index Mark III (HUI3) instrument. The QALYs calculation based on SF-6D utility scores [55] was a special feature of this model, instead of conventional input of utility estimates elicited from conventional direct valuation methods or measured by HUI3. In addition, utility scores for non-CRC (or cancer free) health states were not assumed to be one in a majority of past models [16, 19, 64, 72], specifying at value ranging from 0.90 to 0.94 while the rest of models assumed the non-cancer states to be full health with a utility score being one [18, 60]. However, utility scores for non-CRC states (diversified to normal colonic epithelium, low-risk polyp, and high-risk polyp) were no longer assumed to be identical in current study while same utility scores had assumed for both undiagnosed and diagnosed colorectal polyps or CRC [16, 18, 19, 60, 64, 72]. The differentials of cost per QALYs gained were partly explained by the considerable differences in the data source with respect to utility scores for relevant health states.

The consideration of screening strategy has been limited to single strategy, instead of the hybrid strategy with the combination of I-FOBT with colonoscopy or flexible sigmoidoscopy. Theoretically, the hybrid strategy that advocates the complementary implementation of annual I-FOBT and colonoscopy every 10 years is the most effective strategy relative to all competing strategies adopted in current study, but the complicated administration and delivery of such screening strategy is not executed in an underway large randomized controlled trial [10] that was assigned to either one-time colonoscopy or biennial I-FOBT. Challenges still remained in overcoming the practical concerns over the logistic delivery of hybrid strategy in real world situation.

Several limitations with respect to the model assumptions should be noted. First, our results were primarily simulated by Markov modeling. We assumed that the disease progression and cost spending were the same in the tumour locations of colon and rectum. It is believed that the incidence and mortality rates for colon cancer were overall greater than those for rectal cancer but the direct medical expenditures for colon cancer were cheaper than those for rectal cancer. Adjustment for tumour site could yield simulation results in a more precise way. Second, the utility data was measured by cross-sectional study rather than randomized controlled trial with sufficient follow-up periods, which involves the consideration of time-dependent utility data in the short and long term.

This health economic evaluation has informed the clinicians and policy makers that I-FOBT every one or two years emerged as the most effective and cost-effective colorectal cancer screening strategy compared with no screening in Chinese population. The uncertainty analysis surrounding the major parameters supported the cost-effectiveness analysis derived from base-case scenario. Strategies that utilized colonoscopy alone and annual G-FOBT alone were dominated by other currently recommended strategies for population-based screening. The findings were generalizable to Chinese population, as the cost and clinical parameters input were mostly based on Chinese data. Despite no reaching consensus, such conclusion recommended the inclusion of I-FOBT to the guidelines on colorectal cancer screening for Chinese population.

## Conclusion

The Markov model informed the health policymakers that I-FOBT every year may be the most effective and cost-effective CRC screening strategy among recommended screening strategies, depending on the willingness-to-pay of mass screening for Chinese population.

## Additional file

Additional file 1: **Appendix A.** Natural History Parameters, Performance Characteristics and Compliance Rate of the G-FOBT, I-FOBT and Colonoscopy Used in Markov Model. **Appendix B.** Model Validation Results. **Appendix C.** Costs Parameters and Utility Scores by Stage of Colorectal Neoplasms Used in the Markov Model. **Appendix D.** Cut-off values Used in the Univariate Sensitivity Analysis and Probability Distributions with Associated Distribution Parameters of Model Parameters Used in Probabilistic Sensitivity Analysis. (DOCX 91 kb)

## Abbreviations

CRC: Colorectal cancer
CEA: Cost-effectiveness analysis
G-FOBT: Guaiac fecal occult blood testing
I-FOBT: Immunologic fecal occult blood testing
QALYs: Quality-adjusted life-years
ICER: Incremental cost-effectiveness ratio
LY: Life-years
RCT: Randomized controlled trial
CRN: Colorectal neoplasms
NICE: National Centre for Clinical Excellence
PrSA: Probabilistic sensitivity analysis
HUI3: Health Utility Index Mark III

## References

[1] Shaukat A, Mongin SJ, Geisser MS, Lederle FA, Bond JH, Mandel JS, Church TR (2013). Long-Term Mortality after Screening for Colorectal Cancer. N Engl J Med.

[2] Rex DK, Johnson DA, Anderson JC, Schoenfeld PS, Burke CA, Inadomi JM (2009). American College of Gastroenterology Guidelines for Colorectal Cancer Screening 2008. Am J Gastroenterol.

[3] Muller AD, Sonnenberg A (1995). Protection by Endoscopy Against Death From Colorectal Cancer: A Case–control Study Among Veterans. Arch Intern Med.

[4] Baxter NN, Goldwasser MA, Paszat LF, Saskin R, Urbach DR, Rabeneck L (2009). Association of Colonoscopy and Death From Colorectal Cancer. Ann Intern Med.

[5] Mandel JS, Church TR, Bond JH, Ederer F, Geisser MS, Mongin SJ, Snover DC, Schuman LM (2000). The Effect of Fecal Occult-Blood Screening on the Incidence of Colorectal Cancer. N Engl J Med.

[6] Mandel JS, Church TR, Ederer F, Bond JH (1999). Colorectal Cancer Mortality: Effectiveness of Biennial Screening for Fecal Occult Blood. J Natl Cancer Inst.

[7] Kronborg O, Fenger C, Olsen J, Jørgensen OD, Søndergaard O (1996). Randomised study of screening for colorectal cancer with faecal-occult-blood test. Lancet.

[8] Hardcastle JD, Chamberlain JO, Robinson MHE, Moss SM, Amar SS, Balfour TW, James PD, Mangham CM (1996). Randomised controlled trial of faecal-occult-blood screening for colorectal cancer. Lancet.

[9] Mandel JS, Bond JH, Church TR, Snover DC, Bradley GM, Schuman LM, Ederer F, The Minnesota Colon Cancer Control Study (1993). Reducing Mortality from Colorectal Cancer by Screening for Fecal Occult Blood. N Engl J Med.

[10] Quintero E, Castells A, Bujanda L, Cubiella J, Salas D, Lanas Á, Andreu M, Carballo F, Morillas JD, Hernández C (2012). Colonoscopy versus Fecal Immunochemical Testing in Colorectal-Cancer Screening. N Engl J Med.

[11] Frazier AL, Colditz GA, Fuchs CS, Kuntz KM (2000). Cost-effectiveness of Screening for Colorectal Cancer in the General Population. JAMA.

[12] Zauber AG, Lansdorp-Vogelaar I, Knudsen AB, Wilschut J, van Ballegooijen M, Kuntz KM (2008). Evaluating Test Strategies for Colorectal Cancer Screening: A Decision Analysis for the U.S. Preventive Services Task Force. Ann Intern Med.

[13] Tsoi KKF, Ng SSM, Leung MCM, Sung JJY (2008). Cost-effectiveness analysis on screening for colorectal neoplasm and management of colorectal cancer in Asia. Aliment Pharmacol Ther.

[14] Woo PP, Kim JJ, Leung GM, Woo PPS, Kim JJ, Leung GM (2007). What is the most cost-effective population-based cancer screening program for Chinese women?. J Clin Oncol.

[15] National Institute for Clinical Excellence (2008). Guide to the Methods of Technology Appraisal (reference N1618).

[16] Tappenden P, Chilcott J, Eggington S, Sakai H, Karnon J, Patnick J (2007). Option appraisal of population-based colorectal cancer screening programmes in England. Gut.

[17] Whynes DK, Neilson AR, Walker AR, Hardcastle JD (1998). Faecal occult blood screening for colorectal cancer: is it cost-effective?. Health Econ.

[18] Telford JJ, Levy AR, Sambrook JC, Zou D, Enns RA (2010). The cost-effectiveness of screening for colorectal cancer. CMAJ.

[19] Sharaf RN, Ladabaum U (2013). Comparative Effectiveness and Cost-Effectiveness of Screening Colonoscopy vs. Sigmoidoscopy and Alternative Strategies. Am J Gastroenterol.

[20] Sung JJY, Lau JYW, Goh KL, Leung WK (2005). Increasing incidence of colorectal cancer in Asia: implications for screening. Lancet Oncol.

[21] Sonnenberg FA, Beck JR (1993). Markov Models in Medical Decision Making: A Practical Guide. Med Decis Mak.

[22] Sung JJY, Chan FKL, Leung WK, Wu JCY, Lau JYW, Ching J, To KF, Lee YT, Luk YW, Kung NNS (2003). Screening for colorectal cancer in Chinese: Comparison of fecal occult blood test, flexible sigmoidoscopy, and colonoscopy. Gastroenterology.

[23] Atkin WS, Saunders BP (2002). Surveillance guidelines after removal of colorectal adenomatous polyps. Gut.

[24] Greene FL, Page DL, Fleming ID, Balch CM, Haller DG, Morrow M (2002). AJCC Cancer Staging Manual.

[25] Leshno M, Halpern Z, Arber N (2003). Cost-Effectiveness of Colorectal Cancer Screening in the Average Risk Population. Health Care Manag Sci.

[26] SEER Cancer Statistics Review, 1975–2007 [http://seer.cancer.gov/csr/1975_2007/]. Accessed 28 March 2014.

[27] Hong Kong Cancer Registry Web Site [http://www.ha.org.hk/cancereg]. Accessed 28 March 2014.

[28] Hong Kong Census and Statistics Department (2007). Projected Hong Kong life tables, 2007–2036.

[29] U.S. Preventive Services Task Force (2008). Screening for Colorectal Cancer: U.S. Preventive Services Task Force Recommendation Statement. Ann Intern Med.

[30] Macafee DAL, Waller M, Whynes DK, Moss S, Scholefield JH (2008). Population screening for colorectal cancer: the implications of an ageing population. Br J Cancer.

[31] Lisi D, Hassan CC, Crespi M (2010). Participation in colorectal cancer screening with FOBT and colonoscopy: An Italian, multicentre, randomized population study. Dig Liver Dis.

[32] Hol L, Van Leerdam ME, Van Ballegooijen M, Van Vuuren AJ, Van Dekken H, Reijerink JCIY, Van der Togt ACM, Habbema JDF, Kuipers EJ (2010). Screening for colorectal cancer: randomised trial comparing guaiac-based and immunochemical faecal occult blood testing and flexible sigmoidoscopy. Gut.

[33] Goulard H, Boussac-Zarebska M, Ancelle-Park R, Bloch J (2008). French colorectal cancer screening pilot programme: results of the first round. J Med Screen.

[34] Weller D, Moss S, Butler P, Campbell C, Coleman D, Melia J, Robertson R (2006). English Pilot of Bowel Cancer Screening: an evaluation of the second round. Final Report to the Department of Health.

[35] Newcomb PA, Storer BE, Morimoto LM, Templeton A, Potter JD (2003). Long-Term Efficacy of Sigmoidoscopy in the Reduction of Colorectal Cancer Incidence. J Natl Cancer Inst.

[36] Wong BC, Chan AO, Wong KW, Ching CK, Wong WM, Tam S, Lai KC, Chan CK, Yuen MF, Lam SK (2005). A pilot study of participation in faecal occult blood testing and screening colonoscopy after health education in Hong Kong. Eur J Cancer Prev.

[37] Wong WM, Lam SK, Cheung KL, Tong TSM, Rozen P, Young GP, Chu KW, Ho J, Law WL, Tung HM (2003). Evaluation of an automated immunochemical fecal occult blood test for colorectal neoplasia detection in a Chinese population. Cancer.

[38] Whitlock EP, Lin JS, Liles E, Beil TL, Fu R (2008). Screening for Colorectal Cancer: A Targeted, Updated Systematic Review for the U.S. Preventive Services Task Force. Ann Intern Med.

[39] Macrae FA, Tan KG, Williams CB (1983). Towards safer colonoscopy: a report on the complications of 5000 diagnostic or therapeutic colonoscopies. Gut.

[40] Levin TR, Conell C, Shapiro JA, Chazan SG, Nadel MR, Selby JV (2002). Complications of screening flexible sigmoidoscopy. Gastroenterology.

[41] Segnan N, Senore C, Andreoni B, Aste H, Bonelli L, Crosta C, Ferraris R, Gasperoni S, Penna A, Risio M (2002). Baseline Findings of the Italian Multicenter Randomized Controlled Trial of “Once-Only Sigmoidoscopy”--SCORE. J Natl Cancer Inst.

[42] Nelson DB, McQuaid KR, Bond JH, Lieberman DA, Weiss DG, Johnston TK (2002). Procedural success and complications of large-scale screening colonoscopy. Gastrointest Endosc.

[43] Gatto NM, Frucht H, Sundararajan V, Jacobson JS, Grann VR, Neugut AI (2003). Risk of Perforation After Colonoscopy and Sigmoidoscopy: A Population-Based Study. J Natl Cancer Inst.

[44] Levin TR, Zhao W, Conell C, Seeff LC, Manninen DL, Shapiro JA, Schulman J (2006). Complications of Colonoscopy in an Integrated Health Care Delivery System. Ann Intern Med.

[45] Rathgaber SW, Wick TM (2006). Colonoscopy completion and complication rates in a community gastroenterology practice. Gastrointest Endosc.

[46] Ko CW, Riffle S, Shapiro JA, Saunders MD, Lee SD, Tung BY, Kuver R, Larson AM, Kowdley KV, Kimmey MB (2007). Incidence of minor complications and time lost from normal activities after screening or surveillance colonoscopy. Gastrointest Endosc.

[47] Weinstein MC, O’Brien B, Hornberger J, Jackson J, Johannesson M, McCabe C, Luce BR (2003). Principles of Good Practice for Decision Analytic Modeling in Health-Care Evaluation: Report of the ISPOR Task Force on Good Research Practices—Modeling Studies. Value Health.

[48] Brenner H, Chang-Claude J, Seiler CM, Rickert A, Hoffmeister M (2011). Protection From Colorectal Cancer After Colonoscopy: A Population-Based, Case–Control Study. Ann Intern Med.

[49] Wong CKH, Lam CLK, Poon JTC, McGhee SM, Law WL, Kwong DLW, Tsang J, Chan P (2012). Direct Medical Costs of Care for Chinese Patients with Colorectal Neoplasia: a Health Care Service Provider Perspective. J Eval Clin Pract.

[50] Cheng KC, Yeung YP, Lau PYY, Meng WCS (2008). Surveillance and outcome of liver metastasis in patients with colorectal cancer who had undergone curative-intent operation. Hong Kong Med J.

[51] Hospital Authority. List of charges: S.S. No. 4 to Gazette No. 44/1996 and G.N. 2028 to Gazette No. 13/2003. Hong Kong: Hong Kong Government Printers; 1996, 2003.

[52] Lam CLK, Brazier J, McGhee SM (2008). Valuation of the SF-6D Health States Is Feasible, Acceptable, Reliable, and Valid in a Chinese Population. Value Health.

[53] McGhee SM, Brazier J, Lam CLK, Wong LC, Chau J, Cheung A, Ho A (2011). Quality-adjusted life years: population-specific measurement of the quality component. Hong Kong Med J.

[54] Wong CKH, Mulhern B, Wan Y-F, Lam CLK (2014). Responsiveness was similar between direct and mapped SF-6D in colorectal cancer patients who declined. J Clin Epidemiol.

[55] Wong CKH, Lam CLK, Poon JTC, Kwong DLW (2013). Clinical Correlates of Health Preference and Generic Health-related Quality of Life in Patients with Colorectal Neoplasms. PLoS One.

[56] Cantor SB (1994). Cost - Effectiveness Analysis, Extended Dominance, and Ethics. Med Decis Mak.

[57] Barton GR, Briggs AH, Fenwick EAL (2008). Optimal Cost-Effectiveness Decisions: The Role of the Cost-Effectiveness Acceptability Curve (CEAC), the Cost-Effectiveness Acceptability Frontier (CEAF), and the Expected Value of Perfection Information (EVPI). Value Health.

[58] Stinnett AA, Mullahy J (1998). Net Health Benefits: a new framework for the analysis of uncertainty in cost-effectiveness analysis. Med Decis Mak.

[59] Wilschut JA, Steyerberg EW, van Leerdam ME, Lansdorp-Vogelaar I, Habbema JDF, van Ballegooijen M (2011). How much colonoscopy screening should be recommended to individuals with various degrees of family history of colorectal cancer?. Cancer.

[60] Dan YY, Chuah BYS, Koh DCS, Yeoh KG (2012). Screening Based on Risk for Colorectal Cancer Is the Most Cost-Effective Approach. Clin Gastroenterol Hepatol.

[61] Vijan S, Hwang I, Inadomi J, Wong RKH, Choi JR, Napierkowski J, Koff JM, Pickhardt PJ (2007). The Cost-Effectiveness of CT Colonography in Screening for Colorectal Neoplasia. Am J Gastroenterol.

[62] Ladabaum U, Phillips KA (2006). Colorectal Cancer Screening: Differential Costs for Younger Versus Older Americans. Am J Prev Med.

[63] Song K, Fendrick AM, Ladabaum U (2004). Fecal DNA testing compared with conventional colorectal cancer screening methods: a decision analysis. Gastroenterology.

[64] Sharp L, Tilson L, Whyte S, O’Ceilleachair A, Walsh C, Usher C, Tappenden P, Chilcott J, Staines A, Barry M (2012). Cost-effectiveness of population-based screening for colorectal cancer: a comparison of guaiac-based faecal occult blood testing, faecal immunochemical testing and flexible sigmoidoscopy. Br J Cancer.

[65] Briggs AH (2000). Handling Uncertainty in Cost-Effectiveness Models. PharmacoEconomics.

[66] Berchi C, Bouvier V, Réaud J-M, Launoy G (2004). Cost-effectiveness analysis of two strategies for mass screening for colorectal cancer in France. Health Econ.

[67] van Rossum LGM, van Rijn AF, Verbeek ALM, van Oijen MGH, Laheij RJF, Fockens P, Jansen JBMJ, Adang EMM, Dekker E (2011). Colorectal cancer screening comparing no screening, immunochemical and guaiac fecal occult blood tests: A cost-effectiveness analysis. Int J Cancer.

[68] Lejeune C, Dancourt V, Arveux P, Bonithon-Kopp C, Faivre J (2010). Cost-effectiveness of screening for colorectal cancer in France using a guaiac test versus an immunochemical test. Int J Technol Assess Health Care.

[69] Pignone MP, Flitcroft KL, Howard K, Trevena LJ, Salkeld GP, John DJBS (2011). Costs and cost-effectiveness of full implementation of a biennial faecal occult blood test screening program for bowel cancer in Australia. MJA.

[70] O’Leary BA, Olynyk JK, Neville AM, Platell CF (2004). Cost-effectiveness of colorectal cancer screening: Comparison of community-based flexible sigmoidoscopy with fecal occult blood testing and colonoscopy. J Gastroenterol Hepatol.

[71] Graves N, McKinnon L, Leggett B, Newman B (2005). Re-interpreting the data on the cost and effectiveness of population screening for colorectal cancer in Australia. Aust New Zealand Health Policy.

[72] Heitman SJ, Hilsden RJ, Au F, Dowden S, Manns BJ (2010). Colorectal cancer screening for average-risk North Americans: an economic evaluation. PLoS Med.

[73] Ness RM, Holmes AM, Klein R, Dittus R (1999). Utility valuations for outcome states of colorectal cancer. Am J Gastroenterol.

