# Supplementary material for: Cost-effectiveness simulation and analysis of colorectal cancer screening in Hong Kong Chinese population: comparison amongst colonoscopy, guaiac and immunologic fecal occult blood testing
Source: BMC Cancer. 2015 Oct 15;15:705. doi: 10.1186/s12885-015-1730-y (PMC4608156; doi:10.1186/s12885-015-1730-y)
Supplement: Additional file 1: — Appendix A. Natural History Parameters, Performance Characteristics and Compliance Rate of the G-FOBT, I-FOBT and Colonoscopy Used in Markov Model. Appendix B. Model Validation Results. Appendix C. Costs Parameters and Utility Scores by Stage of Colorectal Neoplasms Used in the Markov Model. Appendix D. Cut-off values Used in the Univariate Sensitivity Analysis and Probability Distributions with Associated Distribution Parameters of Model Parameters Used in Probabilistic Sensitivity Analysis. (DOCX 91 kb) [file 12885_2015_1730_MOESM1_ESM.docx]

**Cost-effectiveness simulation and analysis of colorectal cancer screening in Hong Kong Chinese population: comparison amongst colonoscopy, guaiac and immunologic fecal occult blood testing**

Additional file 1

| Appendix A. Natural History Parameters, Performance Characteristics and Compliance Rate of the G-FOBT, I-FOBT and Colonoscopy Used in Markov Model | | | | | | | | | | | | |  |  |
| --- | --- | --- | --- | --- | --- | --- | --- | --- | --- | --- | --- | --- | --- | --- |
|  | | | | | |  | | | |  | | |  |  |
| Natural History | | | | | | Base-case | | | | Reference | | |  |  |
| **Prevalence** | | | | | |  | | | |  | | |  |  |
| Normal | | | | | | 69.90% | | | | ([Sung *et al*, 2003](#_ENREF_18)) | | |  |  |
| Low-risk polyp | | | | | | 17.62% | | | | ([Sung *et al*, 2003](#_ENREF_18)) | | |  |  |
| High-risk polyp | | | | | | 11.68% | | | | ([Sung *et al*, 2003](#_ENREF_18)) | | |  |  |
| CRC | | | | | | 0.79% | | | | ([Sung *et al*, 2003](#_ENREF_18)) | | |  |  |
|  | | | | | |  | | | |  | | |  |  |
| **Probability of symptomatic presentation (detection without screening)** | | | | | |  | | | |  | | |  |  |
| Low-risk polyps | | | | | | 0% | | | | Assumption | | |  |  |
| High-risk polyps | | | | | | 0% | | | | Assumption | | |  |  |
| CRC Stage I | | | | | | 20% | | | | ([Altekruse *et al*, 2010](#_ENREF_1)) | | |  |  |
| CRC Stage II | | | | | | 20% | | | | ([Altekruse *et al*, 2010](#_ENREF_1)) | | |  |  |
| CRC Stage III | | | | | | 65% | | | | ([Altekruse *et al*, 2010](#_ENREF_1)) | | |  |  |
| CRC Stage IV | | | | | | 100% | | | | ([Altekruse *et al*, 2010](#_ENREF_1)) | | |  |  |
|  | | | | | |  | | | |  | | |  |  |
| **Annual Transition Probability** | | | | | |  | | | |  | | |  |  |
| from normal to low-risk polyps | | | | | | 1.60% | | | | ([Tappenden *et al*, 2007](#_ENREF_19)) | | |  |  |
| from low-risk polyps to high-risk polyps | | | | | | 1.67% | | | | ([Frazier *et al*, 2000](#_ENREF_2); [Hur *et al*, 2007](#_ENREF_6); [Parekh *et al*, 2008](#_ENREF_14)) | | |  |  |
| from high-risk polyps to CRC Stage I | | | | | | 3.26% | | | | ([Tappenden *et al*, 2007](#_ENREF_19)) | | |  |  |
| from high-risk polyps to CRC Stage II | | | | | | 1.74% | | | | ([Frazier *et al*, 2000](#_ENREF_2); [Tappenden *et al*, 2007](#_ENREF_19)) | | |  |  |
| from CRC Stage I to CRC Stage II | | | | | | 30% | | | | ([Pickhardt *et al*, 2007](#_ENREF_15)) | | |  |  |
| from CRC Stage II to CRC Stage III | | | | | | 45% | | | | ([Hur *et al*, 2007](#_ENREF_6)) | | |  |  |
| from CRC Stage III to CRC Stage IV | | | | | | 50% | | | | ([Hur *et al*, 2007](#_ENREF_6)) | | |  |  |
|  | | | | | |  | | | |  | | |  |  |
| **Annual mortality of CRC patients by stage** | | | | | |  | | | |  | | |  |  |
| CRC Stage I | | | | | | 0% | | | | ([Tsoi *et al*, 2008](#_ENREF_20)) | | |  |  |
| CRC Stage II | | | | | | 1% | | | | ([Tsoi *et al*, 2008](#_ENREF_20)) | | |  |  |
| CRC Stage III | | | | | | 6% | | | | ([Tsoi *et al*, 2008](#_ENREF_20)) | | |  |  |
| CRC Stage IV | | | | | | 38.7% | | | | ([Tsoi *et al*, 2008](#_ENREF_20)) | | |  |  |
| Performance Characteristics | | | | | | Base-case | | | | Reference | | |  |  |
| **G-FOBT** | | | | | |  | | | |  | | |  |  |
| Sensitivity for polyps and cancer | | | | | | 19.1% | | | | ([Sung *et al*, 2003](#_ENREF_18)) | | |  |  |
| Specificity for polyps and cancer | | | | | | 79.6% | | | | ([Sung *et al*, 2003](#_ENREF_18)) | | |  |  |
|  | | | | | |  | | | |  | | |  |  |
| **I-FOBT** | | | | | |  | | | |  | | |  |  |
| Sensitivity for polyps and cancer | | | | | | 62.0% | | | | ([Wong *et al*, 2003](#_ENREF_23)) | | |  |  |
| Specificity for polyps and cancer | | | | | | 93.0% | | | | ([Wong *et al*, 2003](#_ENREF_23)) | | |  |  |
|  | | | | | |  | | | |  | | |  |  |
| **Colonoscopy** | | | | | |  | | | |  | | |  |  |
| Sensitivity for polyps and cancer | | | | | | 100.00% | | | | Assumption | | |  |  |
| Specificity for polyps and cancer | | | | | | 100.00% | | | | Assumption | | |  |  |
| Probability of complications | | | | | |  | | | |  | | |  |  |
| Perforation | | | | | | 0.1296% | | | | ([Gatto *et al*, 2003](#_ENREF_3); [Ko *et al*, 2007](#_ENREF_7); [Levin *et al*, 2006](#_ENREF_9); [Macrae *et al*, 1983](#_ENREF_10); [Nelson *et al*, 2002](#_ENREF_13); [Rathgaber & Wick, 2006](#_ENREF_16); [Segnan *et al*, 2002](#_ENREF_17)) | | |  |  |
| Death after perforation | | | | | | 5% | | | | ([Gatto *et al*, 2003](#_ENREF_3); [Rathgaber & Wick, 2006](#_ENREF_16); [Segnan *et al*, 2002](#_ENREF_17)) | | |  |  |
| Bleeding | | | | | | 0.3774% | | | | ([Ko *et al*, 2007](#_ENREF_7); [Levin *et al*, 2006](#_ENREF_9); [Macrae *et al*, 1983](#_ENREF_10); [Nelson *et al*, 2002](#_ENREF_13); [Rathgaber & Wick, 2006](#_ENREF_16)) | | |  |  |
| Death after bleeding | | | | | | 1.4706% | | | | ([Levin *et al*, 2006](#_ENREF_9); [Nelson *et al*, 2002](#_ENREF_13); [Rathgaber & Wick, 2006](#_ENREF_16)) | | |  |  |
| Compliance Rate | | | | | | Base-case | | | | Reference | | |  |  |
| **Screening Interventions** | | | | | |  | | | |  | | |  |  |
| G-FOBT | | | | | | 60% | | | | ([Frazier *et al*, 2000](#_ENREF_2)) | | |  |  |
| I-FOBT | | | | | | 60% | | | | ([Frazier *et al*, 2000](#_ENREF_2)) | | |  |  |
| Colonoscopy | | | | | | 60% | | | | ([Frazier *et al*, 2000](#_ENREF_2)) | | |  |  |
| **Follow-up colonoscopy** | | | | | |  | | | |  | | |  |  |
| given G-FOBT test-positive | | | | | | 80% | | | | ([Frazier *et al*, 2000](#_ENREF_2)) | | |  |  |
| given I-FOBT test-positive | | | | | | 80% | | | | ([Frazier *et al*, 2000](#_ENREF_2)) | | |  |  |
|  | | | | | |  | | | |  | | |  |  |
| **Surveillance Colonoscopy** | | | | | | 80% | | | | ([Frazier *et al*, 2000](#_ENREF_2)) | | |  |  |
| Note: CRC, Colorectal Cancer; G-FOBT, Guaiac fecal occult blood testing; I-FOBT, immunologic fecal occult blood testing | | | | | | | | | | | | |  |  |
| * Colorectal cancer staging will be based on AJCC staging system. | | | | | | | | | | | | |  |  |
|  | | | | | | | | | | | | |  |  |
| Appendix B. Model Validation Results | | | | |  | | |  | |  | | |  | |
|  | |  |  |  | | CRC Mortality Reduction* | | | | | |  | | |
| Screening Strategy/Data | | | Length |  | | Model | | | Literature | | | Reference | | |
| **Biennial G-FOBT** | | |  |  | |  | | |  | | |  | | |
|  | | Funen Trial Data | 10 years |  | | 18.20% | | | 18.00% | | | ([Kronborg *et al*, 1996](#_ENREF_8)) | | |
|  | | Nottingham Trial Data | 13 years |  | | 21.14% | | | 5.66% | | | ([Hardcastle *et al*, 1996](#_ENREF_4)) | | |
|  | | Minnesota Study | 18 years |  | | 26.16% | | | 21.00% | | | ([Mandel *et al*, 2000](#_ENREF_11)) | | |
| **Annual G-FOBT** | | |  |  | |  | | |  | | |  | | |
|  | | Nottingham Trial Data | 13 years |  | | 33.51% | | | 33.41% | | | ([Hardcastle *et al*, 1996](#_ENREF_4)) | | |
|  | | Minnesota Study | 18 years |  | | 40.72% | | | 33.00% | | | ([Mandel *et al*, 2000](#_ENREF_11)) | | |
| Note: CRC, Colorectal Cancer; G-FOBT, Guaiac fecal occult blood testing; I-FOBT, immunologic fecal occult blood testing  * Reduction in mortality rate was calculated as follows: (R_no_screen_ - R_screen_) /R_no_screen_ , where R_screen_ is the mortality rate of a screening strategy and R_no_screen_ is the mortality rate of no screening strategy. | | | | | | | | | | | | | | |

| Appendix C. Costs Parameters and Utility Scores by Stage of Colorectal Neoplasms Used in the Markov Model | | |
| --- | --- | --- |
|  |  |  |
| Unit Cost ($USD) | Base-case | Reference |
| **Cost of Investigation and Follow-up** |  |  |
| General Practitioner Consultation | $28 | Government Gazette([Hospital Authority, 1996, 2003](#_ENREF_5)) |
| Outpatient Specialist Clinic Follow-up | $90 | Government Gazette([Hospital Authority, 1996, 2003](#_ENREF_5)) |
| Carcinoembyonic antigen | $42 | Government Gazette([Hospital Authority, 1996, 2003](#_ENREF_5)) |
|  |  |  |
| **Cost of Screening** |  |  |
| G-FOBT | $4 | Government Gazette([Hospital Authority, 1996, 2003](#_ENREF_5)) |
| I-FOBT | $6 | actim Fecal Blood, Medix Biochemica, Finalnd |
| Colonoscopy with / without biopsy plus Histopathological examination | $1,399 | Government Gazette([Hospital Authority, 1996, 2003](#_ENREF_5)) |
| Bleeding with colonoscopy | $3,320 | ([Tsoi *et al*, 2008](#_ENREF_20)) |
| Perforation with colonoscopy | $10,790 | ([Tsoi *et al*, 2008](#_ENREF_20)) |
|  |  |  |
| **Cost of CRN care in initial phase by stage** |  |  |
| Low-risk Polyps | $1,941 | ([Wong *et al*, 2012](#_ENREF_22)) |
| High-risk Polyps | $5,056 | ([Wong *et al*, 2012](#_ENREF_22)) |
| CRC Stage I | $17,071 | ([Wong *et al*, 2012](#_ENREF_22)) |
| CRC Stage II | $19,755 | ([Wong *et al*, 2012](#_ENREF_22)) |
| CRC Stage III | $26,883 | ([Wong *et al*, 2012](#_ENREF_22)) |
| CRC Stage IV | $45,115 | ([Wong *et al*, 2012](#_ENREF_22)) |
| **Utility** | Base-case | Reference |
| Normal colonic epithelium | 1.000 | Assumption |
| Low-risk polyps | 0.871 | ([Wong *et al*, 2013](#_ENREF_21)) |
| High-risk polyps | 0.827 | ([Wong *et al*, 2013](#_ENREF_21)) |
| CRC Stage I | 0.829 | ([Wong *et al*, 2013](#_ENREF_21)) |
| CRC Stage II | 0.860 | ([Wong *et al*, 2013](#_ENREF_21)) |
| CRC Stage III | 0.814 | ([Wong *et al*, 2013](#_ENREF_21)) |
| CRC Stage IV | 0.738 | ([Wong *et al*, 2013](#_ENREF_21)) |
| **Annual growth rate for cost** | 3.5% | ([National Institute for Clinical Excellence, 2008](#_ENREF_12)) |
| Note: CRC, Colorectal Cancer; G-FOBT, Guaiac fecal occult blood testing; I-FOBT, immunologic fecal occult blood testing | | |
| * Colorectal cancer staging will be based on AJCC staging system. | | |

| Appendix D. Cut-off values Used in the Univariate Sensitivity Analysis and Probability Distributions with Associated Distribution Parameters of Model Parameters Used in Probabilistic Sensitivity Analysis | | | | | | | |
| --- | --- | --- | --- | --- | --- | --- | --- |
|  | | | | | | | |
|  | Base-case | Sensitivity analysis | | Probability distribution | Distribution Parameters | | Reference |
| Model Parameters |  | Low value | High value |  | α | β |  |
| **Natural History of Colorectal Neoplasms** |  |  |  |  |  |  |  |
| **Probability of symptomatic presentation** |  |  |  |  |  |  |  |
| CRC Stage I | 20% | 10% | 30% | Beta | 12 | 48 | Altekruse 2010 |
| CRC Stage II | 20% | 10% | 30% | Beta | 12 | 48 | Altekruse 2010 |
| CRC Stage III | 65% | 45% | 85% | Beta | 14 | 7 | Altekruse 2010 |
| **Transition Probability** |  |  |  |  |  |  |  |
| from normal to low-risk polyps | 1.60% | 0.50% | 5.00% | Beta | 2 | 117 | Tappenden 2007 |
| from low-risk polyps to high-risk polyps | 1.67% | 0.50% | 5.00% | Beta | 2 | 121 | Frazier 2000, Hur 2007, Parekh 2008 |
| from high-risk polyps to CRC Stage I | 3.26% | 0.50% | 5.00% | Beta | 8 | 231 | Tappenden 2007 |
| from high-risk polyps to CRC Stage II | 1.74% | 0.50% | 5.00% | Beta | 2 | 126 | Tappenden 2007, Frazier 2000 |
| from CRC Stage I to CRC Stage II | 30% | 10% | 50% | Beta | 6 | 13 | Chauvin 2011 |
| from CRC Stage II to CRC Stage III | 45% | 25% | 65% | Beta | 10 | 13 | Hur 2007 |
| from CRC Stage III to CRC Stage IV | 50% | 30% | 70% | Beta | 12 | 12 | Hur 2007 |
| **Compliance rate** |  |  |  |  |  |  |  |
| **Screening Interventions** |  |  |  |  |  |  |  |
| G-FOBT | 60% | 20% | 100% | Beta | 3 | 2 | Frazier 2000, Lisi 2010, Fenton 2010 |
| I-FOBT | 60% | 60% | 100% | Beta | 13 | 9 | Frazier 2000, Hol 2010 |
| Sigmoidoscopy | 60% | 30% | 100% | Beta | 4 | 3 | Frazier 2000, Hol 2010 |
| Colonoscopy | 60% | 10% | 100% | Beta | 2 | 1 | Frazier 2000, Lisi 2010 |
| **Follow-up colonoscopy** |  |  |  |  |  |  |  |
| given G-FOBT test-positive | 80% | 80% | 100% | Beta | 48 | 12 | Frazier 2000 |
| given I-FOBT test-positive | 80% | 80% | 100% | Beta | 48 | 12 | Frazier 2000 |
| given Sigmoidoscopy test-positive | 80% | 80% | 100% | Beta | 48 | 12 | Frazier 2000 |
| Survelliance Colonoscopy | 80% | 80% | 100% | Beta | 48 | 12 | Frazier 2000 |
| **Performance Characteristics of G-FOBT** |  |  |  |  |  |  |  |
| Sensitivity for polyps and cancer | 19.1% | 10% | 30% | Beta | 11 | 47 | Sung J, 2003, p.611 |
| Specificity for polyps and cancer | 79.6% | 60% | 100% | Beta | 12 | 3 | Sung J, 2003, p.611 |
| **Performance Characteristics of I-FOBT** |  |  |  |  |  |  |  |
| Sensitivity for polyps and cancer | 62.0% | 40% | 80% | Beta | 13 | 8 | Wong WM, 2003, Table 2 |
| Specificity for polyps and cancer | 93.0% | 70% | 100% | Beta | 9 | 1 | Wong WM, 2003, Table 2 |
| **Probability of complications** |  |  |  |  |  |  |  |
| perforation | 0.1296% | 0.0500% | 0.5000% | Beta | 1 | 980 | Gatto 2003, Levin 2006, Nelson 2002, Rathgaber 2006, Segnan 2002, Macrae 1983, Ko 2007 |
| death after perforation | 5% | 5% | 10% | Beta | 15 | 276 | Gatto 2003, Rathgaber 2006, Segnan 2002 |
| bleeding | 0.3774% | 0.0500% | 0.5000% | Beta | 11 | 2841 | Levin 2006, Nelson 2002, Rathgaber 2006, Macrae 1983, Ko 2007 |
| death after bleeding | 1.4706% | 0.5000% | 5.0000% | Beta | 2 | 107 | Levin 2006, Nelson 2002, Rathgaber 2006 |
| **Utility** |  |  |  |  |  |  |  |
| Low-risk polyps | 0.871 | 0.849 | 0.896 | Beta | 673 | 99 | Wong 2013 |
| High-risk polyps | 0.827 | 0.797 | 0.856 | Beta | 531 | 111 | Wong 2013 |
| Stage I CRC | 0.829 | 0.798 | 0.860 | Beta | 463 | 96 | Wong 2013 |
| Stage II CRC | 0.860 | 0.837 | 0.883 | Beta | 765 | 125 | Wong 2013 |
| Stage III CRC | 0.814 | 0.790 | 0.838 | Beta | 828 | 189 | Wong 2013 |
| Stage IV CRC | 0.738 | 0.705 | 0.772 | Beta | 486 | 172 | Wong 2013 |
| **Annual Discount Rate** | 3.5% | 3% | 5% |  |  |  | NICE guideline 2008 |
|  | Base-case | Sensitivity analysis | |  | Distribution Parameters | | Reference |
| **Unit Cost** |  | Low value | High value |  | µ | σ |  |
| **Cost of Investigation and Follow-up** |  |  |  |  |  |  |  |
| GP consultation | $28 | $26 | $64 | Log normal | 5.31 | 0.35 | HK Government Gazette |
| Outpatient Specialist Clinic Follow-up | $90 | $64 | $ 128 | Log normal | 6.53 | 0.18 | HK Government Gazette |
| Carcinoembyonic antigen | $42 | $38 | $64 | Log normal | 5.79 | 0.15 | HK Government Gazette |
| **Cost of Screening** |  |  |  |  |  |  |  |
| G-FOBT | $ 4 | $ 4 | $ 6 | Log normal | 3.48 | 0.15 | HK Government Gazette |
| I-FOBT | $ 6 | $ 6 | $13 | Log normal | 3.88 | 0.25 | Assumption |
| Sigmoidoscopy with / without biopsy plus Histopathological examination | $ 732 | $ 641 | $1,282 | Log normal | 8.63 | 0.22 | HK Government Gazette |
| Bleeding with sigmoidoscopy | $3,320 | $2,564 | $6,410 | Log normal | 10.12 | 0.29 | Tsoi 2008 |
| Perforation with sigmoidoscopy | $10,790 | $8,974 | $12,821 | Log normal | 11.34 | 0.09 | Tsoi 2008 |
| Colonoscopy with / without biopsy plus Histopathological examination | $1,399 | $1,282 | $1,923 | Log normal | 9.29 | 0.12 | HK Government Gazette |
| Bleeding with colonoscopy | $3,320 | $2,564 | $6,410 | Log normal | 10.12 | 0.29 | Tsoi 2008 |
| Perforation with colonoscopy | $10,790 | $8,974 | $12,821 | Log normal | 11.34 | 0.09 | Tsoi 2008 |
| **Cost of CRC care by stage** |  |  |  |  |  |  |  |
| Low-risk Polyps | $1,941 | $1,748 | $2,232 | Log normal | 9.62 | 0.06 | Wong 2012 |
| High-risk Polyps | $5,056 | $4,767 | $5,248 | Log normal | 10.58 | 0.02 | Wong 2012 |
| Stage I CRC | $17,071 | $14,960 | $20,250 | Log normal | 11.80 | 0.08 | Wong 2012 |
| Stage II CRC | $19,755 | $17,595 | $23,008 | Log normal | 11.94 | 0.07 | Wong 2012 |
| Stage III CRC | $26,883 | $23,638 | $31,770 | Log normal | 12.25 | 0.08 | Wong 2012 |
| Stage IV CRC | $45,115 | $42,899 | $48,453 | Log normal | 12.77 | 0.03 | Wong 2012 |

Reference

Altekruse SF, Kosary CL, Krapcho M, Neyman N, Aminou R, Waldron W, Ruhl J, Howlader N, Tatalovich Z, Cho H, Mariotto A, Eisner MP, Lewis DR, Cronin K, Chen HS, Feuer EJ, Stinchcomb DG, Edwards BK (2010) SEER Cancer Statistics Review, 1975-2007. Bethesda, MD: National Cancer Institute

Frazier AL, Colditz GA, Fuchs CS, Kuntz KM (2000) Cost-effectiveness of Screening for Colorectal Cancer in the General Population. *JAMA* **284**(15)**:** 1954-1961

Gatto NM, Frucht H, Sundararajan V, Jacobson JS, Grann VR, Neugut AI (2003) Risk of Perforation After Colonoscopy and Sigmoidoscopy: A Population-Based Study. *Journal of the National Cancer Institute* **95**(3)**:** 230-236

Hardcastle JD, Chamberlain JO, Robinson MHE, Moss SM, Amar SS, Balfour TW, James PD, Mangham CM (1996) Randomised controlled trial of faecal-occult-blood screening for colorectal cancer. *Lancet* **348**(9040)**:** 1472-1477

Hospital Authority (1996, 2003) List of charges: S.S. No. 4 to Gazette No. 44/1996 and G.N. 2028 to Gazette No. 13/2003. Hong Kong Government Printers

Hur C, Chung DC, Schoen RE, Gazelle GS (2007) The Management of Small Polyps Found by Virtual Colonoscopy: Results of a Decision Analysis. *Clinical Gastroenterology and Hepatology* **5**(2)**:** 237-244

Ko CW, Riffle S, Shapiro JA, Saunders MD, Lee SD, Tung BY, Kuver R, Larson AM, Kowdley KV, Kimmey MB (2007) Incidence of minor complications and time lost from normal activities after screening or surveillance colonoscopy. *Gastrointestinal Endoscopy* **65**(4)**:** 648-656

Kronborg O, Fenger C, Olsen J, JÃ¸rgensen OD, SÃ¸ndergaard O (1996) Randomised study of screening for colorectal cancer with faecal-occult-blood test. *Lancet* **348**(9040)**:** 1467-1471

Levin TR, Zhao W, Conell C, Seeff LC, Manninen DL, Shapiro JA, Schulman J (2006) Complications of Colonoscopy in an Integrated Health Care Delivery System. *Annals of Internal Medicine* **145**(12)**:** 880-886

Macrae FA, Tan KG, Williams CB (1983) Towards safer colonoscopy: a report on the complications of 5000 diagnostic or therapeutic colonoscopies. *Gut* **24**(5)**:** 376-383

Mandel JS, Church TR, Bond JH, Ederer F, Geisser MS, Mongin SJ, Snover DC, Schuman LM (2000) The Effect of Fecal Occult-Blood Screening on the Incidence of Colorectal Cancer. *The New England Journal of Medicine* **343**(22)**:** 1603-1607

National Institute for Clinical Excellence (2008) *Guide to the Methods of Technology Appraisal (reference N1618)*. London: NICE

Nelson DB, McQuaid KR, Bond JH, Lieberman DA, Weiss DG, Johnston TK (2002) Procedural success and complications of large-scale screening colonoscopy. *Gastrointestinal Endoscopy* **55**(3)**:** 307-314

Parekh M, Fendrick AM, Ladabaum U (2008) As tests evolve and costs of cancer care rise: reappraising stool-based screening for colorectal neoplasia. *Alimentary Pharmacology & Therapeutics* **27**(8)**:** 697-712

Pickhardt PJ, Hassan C, Laghi A, Zullo A, Kim DH, Morini S (2007) Cost-effectiveness of colorectal cancer screening with computed tomography colonography. *Cancer* **109**(11)**:** 2213-2221

Rathgaber SW, Wick TM (2006) Colonoscopy completion and complication rates in a community gastroenterology practice. *Gastrointestinal Endoscopy* **64**(4)**:** 556-562

Segnan N, Senore C, Andreoni B, Aste H, Bonelli L, Crosta C, Ferraris R, Gasperoni S, Penna A, Risio M, Rossini FP, Sciallero S, Zappa M, Atkin WS (2002) Baseline Findings of the Italian Multicenter Randomized Controlled Trial of "Once-Only Sigmoidoscopy"--SCORE. *Journal of the National Cancer Institute* **94**(23)**:** 1763-1772

Sung JJY, Chan FKL, Leung WK, Wu JCY, Lau JYW, Ching J, To KF, Lee YT, Luk YW, Kung NNS, Kwok SPY, Li MKW, Chung SCS (2003) Screening for colorectal cancer in Chinese: Comparison of fecal occult blood test, flexible sigmoidoscopy, and colonoscopy. *Gastroenterology* **124**(3)**:** 608-614

Tappenden P, Chilcott J, Eggington S, Sakai H, Karnon J, Patnick J (2007) Option appraisal of population-based colorectal cancer screening programmes in England. *Gut* **56**(5)**:** 677-684

Tsoi KKF, Ng SSM, Leung MCM, Sung JJY (2008) Cost-effectiveness analysis on screening for colorectal neoplasm and management of colorectal cancer in Asia. *Alimentary Pharmacology & Therapeutics* **28**(3)**:** 353-363

Wong CKH, Lam CLK, Poon JTC, Kwong DLW (2013) Clinical Correlates of Health Preference and Generic Health-related Quality of Life in Patients with Colorectal Neoplasms. *PLoS One* **8**(3)**:** e58341

Wong CKH, Lam CLK, Poon JTC, McGhee SM, Law WL, Kwong DLW, Tsang J, Chan P (2012) Direct Medical Costs of Care for Chinese Patients with Colorectal Neoplasia: a Health Care Service Provider Perspective. *Journal of Evaluation in Clinical Practice* **18**(6)**:** 1203-1210

Wong WM, Lam SK, Cheung KL, Tong TSM, Rozen P, Young GP, Chu KW, Ho J, Law WL, Tung HM, Choi HK, Lee YM, Lai KC, Hu WHC, Chan CK, Yuen MF, Wong BC-Y (2003) Evaluation of an automated immunochemical fecal occult blood test for colorectal neoplasia detection in a Chinese population. *Cancer* **97**(10)**:** 2420-2424
